# Mitochondrial plasticity and synaptic plasticity crosstalk; in health and Alzheimer's disease

**DOI:** 10.1111/cns.14897

**Published:** 2024-08-04

**Authors:** Fatemeh Sayehmiri, Fereshteh Motamedi, Zehra Batool, Nima Naderi, Fatima Shaerzadeh, Anahita Zoghi, Omidvar Rezaei, Fariba Khodagholi, Hamid Gholami Pourbadie

**Affiliations:** ^1^ Neuroscience Research Center, Faculty of Medicine Shahid Beheshti University of Medical Sciences Tehran Iran; ^2^ Faculty of Medicine Shahid Beheshti University of Medical Sciences Tehran Iran; ^3^ Dr. Panjwani Center for Molecular Medicine and Drug Research, International Center for Chemical and Biological Sciences University of Karachi Karachi Pakistan; ^4^ Department of Pharmacology and Toxicology, Faculty of Pharmacy Shahid Beheshti University of Medical Sciences Tehran Iran; ^5^ Lacerta Therapeutics Alachua Florida USA; ^6^ Department of Neurology, Loghman Hakim Hospital Shahid Beheshti University of Medical Sciences Tehran Iran; ^7^ Skull Base Research Center Loghman Hakim Hospital, Shahid Beheshti University of Medical Sciences Tehran Iran; ^8^ Department of Physiology and Pharmacology Pasteur Institute of Iran Tehran Iran

**Keywords:** Alzheimer's disease, beta‐amyloid, mitochondria, synaptic plasticity

## Abstract

Synaptic plasticity is believed to underlie the cellular and molecular basis of memory formation. Mitochondria are one of the main organelles involved in metabolism and energy maintenance as plastic organelles that change morphologically and functionally in response to cellular needs and regulate synaptic function and plasticity through multiple mechanisms, including ATP generation, calcium homeostasis, and biogenesis. An increased neuronal activity enhances synaptic efficiency, during which mitochondria's spatial distribution and morphology change significantly. These organelles build up in the pre‐and postsynaptic zones to produce ATP, which is necessary for several synaptic processes like neurotransmitter release and recycling. Mitochondria also regulate calcium homeostasis by buffering intracellular calcium, which ensures proper synaptic activity. Furthermore, mitochondria in the presynaptic terminal have distinct morphological properties compared to dendritic or postsynaptic mitochondria. This specialization enables precise control of synaptic activity and plasticity. Mitochondrial dysfunction has been linked to synaptic failure in many neurodegenerative disorders, like Alzheimer's disease (AD). In AD, malfunctioning mitochondria cause delays in synaptic vesicle release and recycling, ionic gradient imbalances, and mostly synaptic failure. This review emphasizes mitochondrial plasticity's contribution to synaptic function. It also explores the profound effect of mitochondrial malfunction on neurodegenerative disorders, focusing on AD, and provides an overview of how they sustain cellular health under normal conditions and how their malfunction contributes to neurodegenerative diseases, highlighting their potential as a therapeutic target for such conditions.

## Graphical abstract

The mechanism of mitochondrial transport within the neuron is mediated on the cytoskeleton. The retrograde (−) motion deployed the movement of mitochondria toward the cell body with the help of dynein, TRAK1/2, and Mrio1/2 protein. While anterograde (+) motion causes the movement toward the axon with the assistance of kinesin, TRAK1/2, and Miro1/2 proteins. At the axon terminal, the mitochondrion is separated due to the influx of calcium, which destabilizes its complex with kinesin protein. In the cell body, mitochondria undergo fission and fusion process in order to remove its damaged portion or to conserve it, respectively.

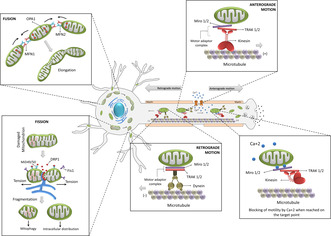

## 1 INTRODUCTION

Controlling and maintaining brain cell metabolism is a crucial challenge for the nervous system. Mitochondrial health is essential for sustaining synaptic function and plasticity in neurons, which have high energy demands due to their complex signaling and maintenance of membrane potentials. Beyond energy production, mitochondria are involved in calcium homeostasis, regulation of apoptosis, and the generation of reactive oxygen species (ROS).^1^ Dysfunctions in these processes can lead to cellular stress and damage. Mitochondrial dysfunction plays a crucial role in the process of aging and the progression of neurodegenerative disorders like Alzheimer's disease (AD).^2^, ^3^, ^4^ Impaired mitochondrial dynamics, including fission, fusion, and transport, contribute to neuronal degeneration and synaptic failure. The accumulation of damaged mitochondria exacerbates oxidative stress and disrupts cellular homeostasis, further accelerating the progression of neurodegenerative conditions.

Mitochondria are considered plastic organelles that change functionally and reshape morphologically in response to cellular needs, named “mitochondrial plasticity.” Growing evidence shows the critical role of mitochondrial plasticity in synaptic transmission, including synthesizing and storing neurotransmitters, synaptic vesicle trafficking, neurotransmitter release at synaptic space, and their further recycling.^5^ Synaptic transmission and plasticity are ATP‐dependent processes that are constructively provided by mitochondria. Moreover, mitochondria take up the excess intracellular Ca^2+^ at the presynaptic terminals to regulate and maintain synaptic transmission or plasticity.^6^, ^7^, ^8^ Besides, balancing ROS production and contributing to the synthesis pathway of several neurotransmitters make these cellular structures essential for synaptic function.^9^, ^10^, ^11^, ^12^, ^13^ Mitochondrial dysfunction and synaptic failure co‐occurrence have been reported in many neurodegenerative diseases, especially AD.^14^, ^15^, ^16^, ^17^

This review explores the crosstalk between mitochondrial plasticity and its association with synaptic plasticity, focusing on mitochondrial dysfunction in AD.

## 2 MITOCHONDRIAL FUNCTION IN SYNAPTIC TRANSMISSION

Proper mitochondrial function largely depends on neurotransmission maintenance in synaptic structures. The critical role of mitochondria in various stages of neurotransmission, including neurotransmitter synthesis and storage, synaptic vesicle (SVs) trafficking, neurotransmitter release from presynaptic terminals, and recycling of SVs, has been well proven.^17^ Energy supply, calcium homeostasis maintenance, and synthesizing essential intermediates or final productions of several neurotransmitters are among the most critical mitochondrial functions supporting synaptic transmission.^9^, ^10^, ^11^, ^12^

### 2.1 Mitochondria guarantee neuronal function by calcium buffering

Synaptic communication relies on the fusion of vesicles containing neurotransmitters with the presynaptic membrane, facilitated by the Ca^2+^ influx. The mitochondria, endoplasmic reticulum (ER), and lysosomes are essential organelles in calcium buffering. When synaptic transmission initiates, voltage‐gated Ca^2+^ channels (VGCCs) open secondary to the depolarization of the cellular membrane, enabling a rapid influx of Ca^2+^ into the presynaptic terminal. The fast influx of calcium ions facilitates the fusion of the vesicles with the synaptic membrane, resulting in neurotransmitter release.^18^

Simultaneous with this rapid influx of Ca^2+^ ions, there is a significant increase in Ca^2+^ levels within the synaptic mitochondria, which plays a vital role in synaptic activity.^19^ During this process, mitochondria serve as a calcium buffer by absorbing significant amounts of calcium in response to temporary increases in cytoplasmic calcium levels and storing the calcium in its matrix.^6^, ^20^ Aside from mitochondria, the ER also functions as a reservoir and supplier of calcium, regulating its levels to ensure calcium homeostasis.^21^

Following fractionation studies, Jean Vance coined the term mitochondria‐associated membranes (MAMs) proteins to describe the specific protein domains at the ER membrane that are located in their contact sites with mitochondria and are responsible for lipid metabolism.^22^ MAMs also play a crucial role in maintaining Ca^2+^ balance.^23^, ^24^, ^25^ This is evident from the inositol‐1,4,5‐trisphosphate receptor (IP3R) acting as a Ca^2+^ channel at these sites.^26^, ^27^ The release of Ca^2+^ through IP3Rs from the ER creates local high concentrations of Ca^2+^, which play a pivotal role in the uptake of Ca^2+^ into the matrix of mitochondria.^23^, ^28^ Firstly, Mitochondrial Ca^2+^ uptake occurs through the diffusion of Ca^2+^ across voltage‐dependent anion channels (VDACs) located in the outer membrane of mitochondria.^29^ The Ca^2+^ is then taken up by the mitochondrial calcium uniporter (MCU), which is considered low affinity compared to VDAC and located close to the inner membrane of mitochondria.^30^, ^31^ The MCU is a normally closed Ca^2+^‐selective ion channel that opens in response to cytosolic Ca^2+^ elevation,^31^, ^32^, ^33^ which mediates their Ca^2+^‐dependent activation.^33^ The physiologic elevation of mitochondrial Ca^2+^ through the MCU can increase oxidative phosphorylation, regulate synapses, and stimulate local ATP generation in presynaptic terminals. The mitochondrial uptake of Ca^2+^ can also influence the amount and spatiotemporal dynamics of cytosolic Ca^2+^, consequently regulating Ca^2+^‐dependent signaling. Effects of the MCU aside from its physiological function increased mitochondrial Ca^2+^ uptake can interfere with neuronal mortality pending acute excitotoxicity and degenerative illnesses like AD.^34^

Mitochondria, lysosomes, and the endoplasmic reticulum (ER) are all implicated, albeit to varying degrees, in regulating synaptic Ca^2+^ concentrations. While the intracellular Ca^2+^ concentration typically ranges from 50 to 100 nM, levels within the ER and lysosomes reach the hundreds of micromolar range.^35^ This suggests that Ca^2+^ release from these organelles can influence Ca^2+^ signaling at the presynapse, impacting neuronal activity. Numerous lines of evidence from cells with dysfunctional mitochondria underscore the crosstalk between mitochondria and lysosomes. For instance, mouse embryonic fibroblasts lacking key proteins like AIFM1, essential for respiratory chain function, OPA1, crucial for mitochondrial fusion, or PINK1, involved in respiratory chain quality control and mitophagy, exhibit impaired lysosomal function.^36^ This is manifested by the enlargement of lysosomal vesicles positive for LAMP1, which lose their acidity and hydrolytic activity. The adverse impact of mitochondrial dysfunction on lysosomes is consistent across various cell types.^37^ Notably, this effect appears independent of decreased ATP availability. On the other hand, recent findings indicate that lysosomal dysfunction plays a central role in a transcriptional program that inhibits both mitochondrial biogenesis and function.^38^ Lysosomes are involved in both degradative and signaling pathways at the presynaptic terminal. The local degradative capacity at the presynapse is sustained by the constant supply of lysosomes to distal axonal tips.^39^ This process is essential for recycling or delivering synaptic proteins^40^, ^41^ and removing damaged mitochondria.^40^ Additionally, the volume and organelle distribution at axonal tips also play a role in determining the ER's and lysosomes' contribution to Ca^2+^ signaling.^41^ Lysosomal acidification, crucial for maintaining lysosomal enzyme activity, is closely linked to lysosomal Ca^2+^ levels.^42^ Meanwhile, mitochondria primarily engage in local Ca^2+^ buffering, influencing their ability to provide ATP to sustain the energy demands necessary for neurotransmitter release regulation. Dysfunctions in handling Ca^2+^ by these organelles at the presynapse, mitochondrial energy production, lysosomal waste degradation at axonal tips, or ER‐mediated presynaptic protein synthesis may contribute to neurodegenerative disease development.^41^ This underscores the need for further investigation into these organelles' specific functions and dysfunctions at presynaptic terminals.

Endocannabinoids and purines are two examples of neuromodulators that can directly interact with mitochondria through specific receptors. Cannabinoid receptors, including CB1, are expressed on mitochondrial membranes, which play essential roles in modulating mitochondrial functions activity‐dependently. Activation of mitochondrial CB1 receptors can influence cellular energy metabolism by altering ATP production, affecting reactive oxygen species (ROS) generation, and regulating calcium homeostasis within cells. These effects are highly context dependent and vary based on the cellular environment and specific signaling pathways. Retrograde control of hippocampal GABAergic transmission is a crucial form of synaptic plasticity that depends on endocannabinoids. When CA1 postsynaptic pyramidal neurons are depolarized, endocannabinoids are mobilized and leads to activation of presynaptic CB1 receptors and consequently reduces GABAergic inhibitory neurotransmission, a process known as depolarization‐induced suppression of inhibition (DSI).^43^, ^44^, ^45^ Mitochondrial CB1 receptors are involved in DSI in the hippocampus.^46^ Purinergic receptors are a family of receptors activated by purine nucleotides such as ATP, ADP, UTP, etc. They are divided into two main types: P1 receptors (adenosine receptors) and G protein‐coupled receptors that respond to adenosine, and P2 receptors. Sarti et al.^47^ demonstrated the presence of a substantial functional pool of P2X7 purinoceptors within mitochondria, situated in the outer mitochondrial membrane with their ATP‐binding sites oriented toward the cytosol. These receptors are linked to Complex I via an undiscovered cascade mechanism.^48^ When activated, P2X7 receptors enhance the expression and function of Complex I, thereby boosting mitochondrial polarization. This polarization increases ionized Ca^2+^ levels within the matrix and increases ATP production.^49^ P2X synapse receptors exhibit a higher calcium permeability than glutamate ionotropic receptors.^48^, ^50^ This heightened calcium influx through P2X receptors holds significant sway over synaptotoxicity, potentially surpassing the impact of glutamate signaling. Consequently, pathological alterations in mitochondrial function could result from this excitotoxic neuronal injury. Such changes may manifest as mitochondrial dynamics, bioenergetics, and calcium homeostasis disruptions, contributing to neuronal dysfunction and cell death in various neurological disorders. Additionally, mitochondrial P2Y1‐like and P2Y2 receptors regulate mitochondrial Ca^2+^ uptake via the uniporter. Activation of mitochondrial P2Y1 stimulates Ca^2+^ uptake, whereas activation of mP2Y2 inhibits it. ATP primarily targets mP2Y2 receptors, whereas ADP and AMP stimulate both mP2Y1 and mP2Y2 receptors.^51^, ^52^

Additionally, presynaptic terminals have a higher concentration of mitochondria than other areas of neuronal cells.^53^, ^54^, ^55^ These mitochondria drive neurotransmission by generating ATP and buffering Ca^2+^. Depleting mitochondria from these locations limits synaptic transmission because of inadequate ATP supply or changed Ca^2+^ kinetics during intense synaptic activity.^56^

There is evidence that mitochondrial inhibitors increased the frequency of spontaneous synaptic transmission in hippocampal neurons.^57^ Additionally, mitochondria are essential for producing post‐tetanic potentiation (PTP). Synaptic mitochondria preserve Ca^2+^ homeostasis during tetanic neuronal activation by buffering and delivering excess intracellular Ca^2+^ after stimulation to extend its remaining levels.^20^ This mechanism is assumed to be responsible for preserving and controlling neurotransmission^6^ or specific short‐term synaptic plasticity.^8^ So, in addition to producing energy, mitochondria can secure and regulate intracellular Ca^2+^ levels. Excess intracellular Ca^2+^ is taken up and released by mitochondria at presynaptic terminals to maintain the Ca^2+^ levels.^20^ This shows that synaptic mitochondria guarantee synaptic or neuronal function by calcium buffer.

The synaptic environment is dynamic, and fluctuations in pH can significantly impact cellular processes, including Ca^2+^ homeostasis. Mitochondria help maintain this balance by sequestering excess Ca^2+^ during periods of high synaptic activity.^1^ Changes in synaptic pH affect the mitochondrial membrane electrochemical gradient, influencing mitochondrial Ca^2+^ uptake.^58^, ^59^ Under more acidic conditions, mitochondrial Ca^2+^ intake can be impaired, potentially leading to elevated cytosolic Ca^2+^ levels. This can disrupt normal synaptic function, as Ca^2+^ is a critical second messenger involved in neurotransmitter release, synaptic plasticity, and signal transduction pathways. Conversely, alkaline pH conditions can enhance mitochondrial Ca^2+^ buffering capacity, potentially protecting neurons from excitotoxicity induced by excessive Ca^2+^ influx.^60^ The mitochondria's ability to buffer Ca^2+^ effectively during pH changes is also linked to their role in generating ATP, which fuels various ion pumps and transporters that help restore ionic and pH balance.^61^ Therefore, efficient mitochondrial Ca^2+^ buffering is essential for maintaining synaptic stability and function, particularly in the face of pH fluctuations that accompany various physiological and pathological conditions.

### 2.2 Mitochondria ensure neurotransmission by supporting vesicle recycling, synthesis, and storage of neurotransmitter

Mitochondria are essential for vesicle recycling in neurons, which is critical in maintaining synaptic function.^62^ Neuronal communication depends on the rapid and continuous release of neurotransmitters from synaptic vesicles into the synaptic cleft. After neurotransmitter release, these vesicles must be quickly recycled and refilled to sustain synaptic activity.^63^ Mitochondria provide the necessary ATP for this energy‐intensive process, powering the endocytosis machinery to retrieve and refill vesicles with neurotransmitters. This energy supply is crucial for remodeling vesicle membranes and maintaining the proton gradient essential for neurotransmitter loading.^64^ Also, mitochondria help regulate intracellular calcium levels, vital for vesicle fusion and release.^65^ Mitochondrial dysfunction can impair vesicle recycling, leading to defective synaptic transmission and contributing to neurodegenerative diseases, where synaptic failure is a hallmark. Thus, mitochondria's role in vesicle recycling highlights their importance in maintaining neuronal communication and overall brain health.

Mitochondria also play a significant role in synthesizing and storing neurotransmitters necessary for effective neural communication.^66^ As the cell's powerhouses, mitochondria produce ATP required for the biosynthesis of neurotransmitters like acetylcholine, dopamine, and serotonin.^17^ They maintain the proton gradient crucial for loading neurotransmitters into synaptic vesicles, enabling active transport and preparing them for synaptic transmission. Mitochondria are involved in synthesizing precursor molecules needed for neurotransmitter production. For instance, dopamine synthesis requires L‐DOPA, produced through mitochondrial metabolic pathways, and acetyl‐CoA, produced via the Krebs cycle, is necessary for acetylcholine synthesis.^67^, ^68^ Mitochondrial dysfunction can reduce the availability of these precursors, disrupting neurotransmitter synthesis and storage.

Mitochondrial dysfunction is linked to several neurodegenerative diseases due to its impact on neurotransmitter dynamics. In Parkinson's, mitochondrial dysfunction reduces dopamine synthesis, contributing to the disorder's characteristic motor deficits.^69^, ^70^ Similarly, mitochondrial abnormalities in Alzheimer's disease affect acetylcholine levels, leading to cognitive decline.^71^

## 3 MITOCHONDRIAL PLASTICITY AND SYNAPTIC PLASTICITY

Synaptic plasticity causes structural changes within the synapse, such as an increase in the size of the presynaptic active zone, postsynaptic density (PSD), and dendritic spine.^72^ Presynaptic terminals of axons contain a significant number of mitochondria, as the area of the presynaptic terminal active zone is highly correlated to mitochondria volume.^73^ Furthermore, because mitochondria proximity is also independently associated with the amount of neighboring docked vesicles, it is reasonable to conclude that appropriate mitochondrial function is critical for maintaining neurotransmission in synaptic structures.^74^, ^75^ Surprisingly, in a recent study by Thomas et al.,^76^ no relationship was observed between the spine head volume or PSD area, and dendritic mitochondrial volume, showing that presynaptic and postsynaptic distribution of mitochondria does not follow an identical pattern. Furthermore, compared to dendritic mitochondria, presynaptic mitochondria are usually shorter, smaller, and less complicated (Presynaptic mitochondria 0.05 μm^3^ vs. postsynaptic mitochondria 0.195 μm^3^).^77^, ^78^, ^79^ Moreover, as reported by Cserép et al.,^80^ the axons of highly active neurons had larger mitochondria in comparison to low‐activity neurons, suggesting a positive correlation between mitochondrial size and neuronal activity or function. At first glance, it appears that this increase in size is intended to supply more energy.^80^ However, Lewis et al.^81^ reported that enlarged postsynaptic mitochondria did not increase ATP production but augmented their capacity for Ca^2+^ uptake. Consequently, large dendritic mitochondria that span multiple synapses may serve as a substantial reservoir for buffering Ca^2+^ and integrating signals from adjacent synapses. Conversely, small mitochondria in presynaptic terminals may facilitate the rapid elevation of cytoplasmic Ca^2+^ levels necessary to release neurotransmitters.^81^ The half‐life of mitochondria in synapses is typically around 4 weeks.^82^ This duration can vary depending on the specific neuronal context and the metabolic demands placed on the synapse. Presynaptic terminals of axons contain a remarkable amount of mitochondria.^73^ However, neuronal mitochondria have been demonstrated to have a longer half‐life, making them more susceptible to damage, especially their synaptic form, which is usually longer‐lived than others found in the other parts of neurons.^82^, ^83^, ^84^, ^85^, ^86^ Therefore, mitochondrial impairments should be noted since they are common and likely pathologically affect synaptic activity.^9^, ^13^, ^15^, ^87^ Several cases of concomitant mitochondrial dysfunction and synaptic stress have been reported in some neurodegenerative diseases, including AD.^14^, ^15^

**FIGURE 1:**
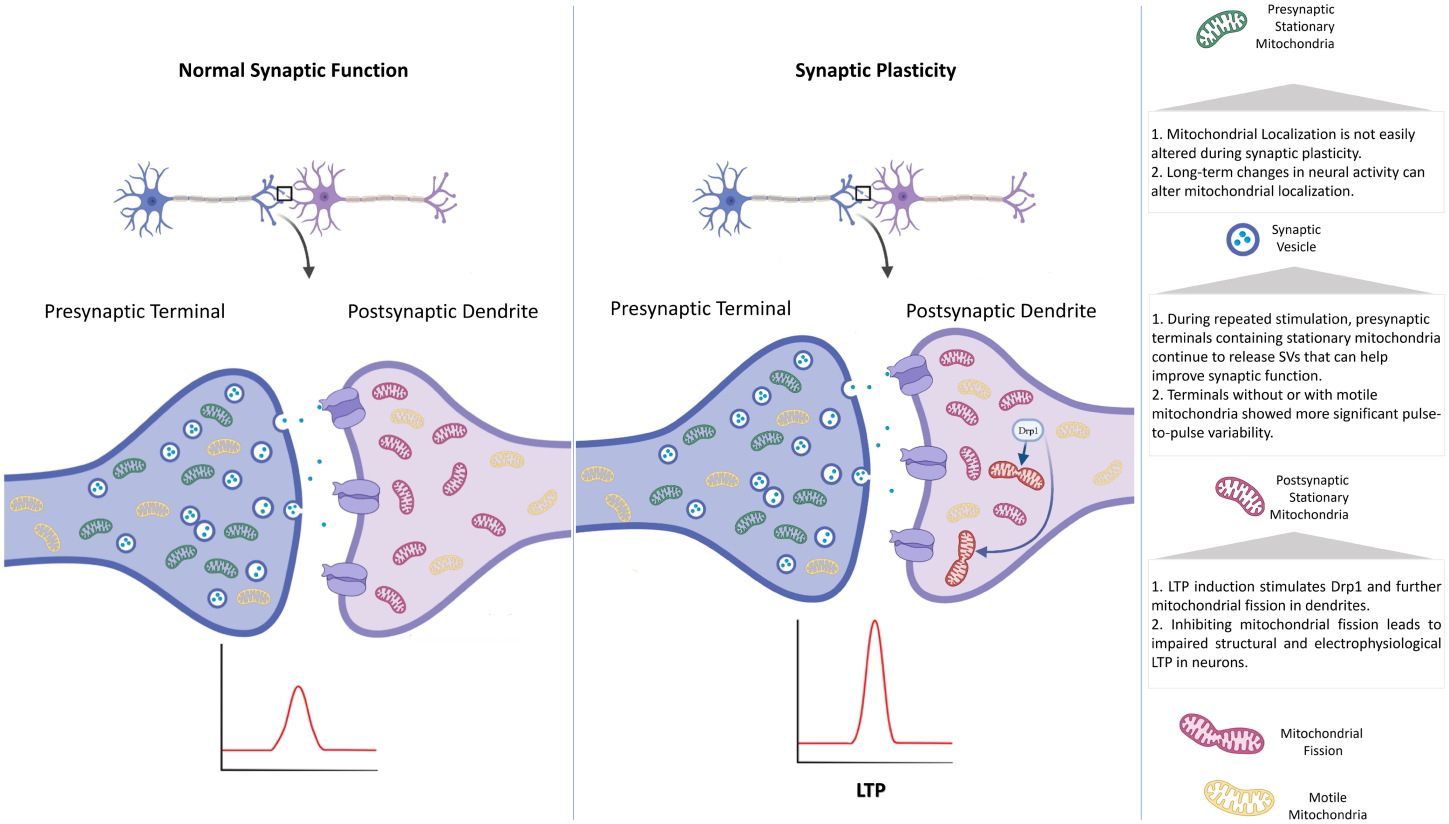
Mitochondria as central hubs in synaptic modulation.

Mitochondria adapt to maintain energy balance and support neuronal functions when energy needs change. This adaptation is called mitochondrial plasticity and can have different manifestations, including alteration in position, size, number, and mitochondrial functions. Under normal conditions, mitochondrial fission and fusion constantly occur to preserve its quality function and establish a coordination between mitochondrial morphology and cellular energy demands. Mitochondrial integrity is supported by the molecular machinery of the fission and fusion process, which is continually performed by regulating gene expression and quality control of protein products.^88^ Previous studies have proven the pivotal pathological role of disruption in mitochondrial dynamics in neurodegenerative disorders. Examples of defective mitochondrial dynamics, respiratory chain dysfunction, imbalance in mitochondrial fission and fusion process, and reduced ATP biogenesis have been observed in pathologies of most neurodegenerative disorders.^89^ As stated earlier, neuron synapses are primary sites of energy depletion, and mitochondria are critical for their proper functions.^90^ In dendritic spines, mitochondrial fission is essential for LTP^91^ (Figure 1).

Most current literature only shows that mitochondrial plasticity and synaptic plasticity co‐occur, but no precise temporal sequence has yet been established. As a result, there is a shortage of extensive discussions or precise data in the existing literature about the priority of one process over another. This knowledge gap emphasizes the importance of future research into the temporal dynamics of mitochondrial and synaptic plasticity, which could reveal critical insights into neural adaptability mechanisms.

### 3.1 Distinct mitochondrial morphology in pre‐ and postsynaptic zones

Mitochondria show different morphology and distribution in the axon and dendrites. Postsynaptic dendritic mitochondria in pyramidal neurons have long, tubular forms occupying roughly one‐third of the dendritic arbors. On the other hand, presynaptic axonal mitochondria are tiny, punctate, and have a strikingly uniform size, occupying 10% of the axonal volume.^92^, ^93^ The possible explanation for the remarkable diversity in mitochondrial morphology between these is increased fission in the axon and fusion in dendrites. It also seems this difference is associated with a change in the functional capacity and output.^94^, ^95^

In response to intracellular signals that control their recruitment into the stationary pool and their velocity, mitochondria are moved to activate synapses. So, mitochondrial plasticity can affect synaptic plasticity at two points:

### 3.2 Mitochondrial plasticity at the presynaptic terminals

Due to intricate mitochondrial anatomy, neurons have specialized within their sub‐compartments to guarantee strict regulation of primary cellular activities.^96^ This complex receives input from numerous signaling channels to customize the mitochondrial location. Mitochondria in neuronal axons are seen in both mobile and stationary forms. The more neurons mature, the more they reach their final position and settle there.^90^, ^97^, ^98^ About half of axonal mitochondria reside at the presynaptic terminals early after birth. Interestingly, this number is 80% in hippocampal neurons of adult rats.^74^ About 10% of the mitochondria were in a state of transit at any given time, whereas the other 90% were immobile or stationary. 40% of these stationary mitochondria remained in place over several days. Using time‐lapse imaging at intervals of 30 min, it was possible to detect and quantitatively analyze the stochastic transition of the remaining mitochondria to the mobile state.^99^ Mitochondria did not stay stationary for longer than a few days. 63.8% of all mitochondria have relocated from their original position. Over 16 days, more than 95% of all mitochondria had either relocated or vanished.^97^ Enhancing mitochondrial motility increases pulse‐to‐pulse variability, while immobilizing mitochondria reduces the variability. During repeated stimulation, presynaptic terminals containing stationary mitochondria keep releasing SVs, enhancing synaptic efficacy.^100^ When the mitochondria move into the high ATP regions, their motility rises. Conversely, their mobility reduces when the mitochondria are adjacent to places with local ATP depletion, such as synapses.^101^ It is noteworthy that mitochondrial reshaping could occur when the demand for quick access to energy increases and eventually provides a plastic metabolic pathway for ATP generation in the presynaptic axon terminal. Mitochondria resident in the presynaptic terminal of glutamatergic neurons and fast‐spiking interneurons developed a favorable morphology and cristae plasticity compared to those in interneurons with slow firing.^80^ The local axonal microenvironment can provide presynaptic terminals with several signals that can regulate mitochondrial function and dynamics, leading to mitochondrial plasticity on demand.^90^ Presynaptic turnover changes are linked to learning and disease, demonstrating how they are crucial for the plasticity of brain circuits.^102^

Synaptic boutons with resident mitochondria have been shown to have more stable synapses than those without. Long‐duration changes in neuronal activity primarily influence the location of mitochondria at presynaptic sites, which is essential for maintaining homeostatic plasticity.^97^ Together, these results imply that most axonal mitochondria reach their destination when neurons develop, and synaptic plasticity processes do not quickly change this. Also, at least one mitochondria in presynaptic boutons increases the number of synaptic vesicles and large synapses.^103^ How can mitochondria lose their ability to move or remain at the presynaptic terminal? Molecular interactions with the axonal cytoskeleton control the distribution and dynamics of mitochondria. The motor adaptor complex, made of Milton and Miro, facilitates mitochondrial trafficking along microtubules.^103^ In vitro research suggests that local variations in Ca^2+^ concentration may affect the distribution of mitochondria throughout the axon. Milton/TRAKs proteins and the Ca^2+^‐binding GTPases Miro1 and Miro2 on the outer mitochondrial membrane mediate mitochondrial binding to motor proteins.^104^ Besides, mitochondrial stalling is caused by changes in Miro1's conformation caused by local Ca^2+^ rise at synapses. These changes may prevent the motor–adaptor interaction or cause Miro1 to detach from microtubules, resulting in mitochondrial trapping in the active synapses.^104^, ^105^ In addition to the cytosolic Ca^2+^ enhancement, when neurons are exposed to glutamate, the neuronal mitochondria show dramatic morphological alterations.^106^ However, neuronal mitochondrial contraction caused by glutamate is also Ca^2+^‐dependent and is linked to the mitochondrial permeability transition.^106^

It has also been shown that glutamatergic synapses are more vulnerable at the early stages of AD,^107^, ^108^, ^109^, ^110^, ^111^ while GABAergic synapses appear to be less affected.^107^, ^112^, ^113^, ^114^ Consequently, it was hypothesized that there is a larger disruption of mitochondria in glutamatergic synapses in comparison with GABAergic synapses. Furthermore, a study conducted on purified hippocampal synapses called synaptosomes from an animal model of AD revealed that exposing these synaptosomes directly to Aβ1–42 resulted in a decrease in mitochondrial membrane potential and an increase in mitochondria‐derived oxygen radicals in glutamatergic and GABAergic synaptosomes. The results suggest different morphology and mitochondria properties are equally changed in glutamatergic and GABAergic terminals in pathologic conditions such as AD.^115^, ^116^

### 3.3 Mitochondrial plasticity at the postsynaptic dendrites

An extended network of mitochondria occupies large areas in dendrites. This unique dendritic mitochondrial distribution develops before synapse formation and stables as the neuron matures. Like axons, selective localization of dendritic mitochondria occurs in dendrites.^117^, ^118^, ^119^ Different studies revealed different results about mitochondrial distribution in other sites of dendrites, like dendritic spines.^120^, ^121^, ^122^ These findings determine synapse‐specific mitochondrial localization in postsynaptic areas.^90^ Accumulating mitochondria in the dendrites boosted the number of spines and synapses while reducing the content of dendritic mitochondria, resulting in synapses and spines losing.^122^ Moreover, recent in vitro investigations revealed that enhanced mitophagy reduces dendritic mitochondrial content, inhibits dendrite growth during neuronal polarization,^123^ and shortens dendrites in mature neuronal cultures.^124^

Throughout development, mitochondrial motility fundamentally alters. Over 30% of mitochondria move at any given time during dendritic growth and synaptogenesis. In contrast, Faits et al. reported no dendritic mitochondrial movement in mature circuits, suggesting that the dominant form of dendritic mitochondria is immobile or stationary. Dendritic mitochondria located in synapses may help with growth and plasticity. Before the formation of the majority of synapses, mitochondria arrive at dendrites to near‐mature levels. The motility of dendritic mitochondria is reduced when external K^+^ is elevated in the retina and cultured neurons. However, neither sensory‐evoked activity during maturity nor spontaneous activity waves during development impacted the mobility of dendritic mitochondria in retinal ganglion cells.

(RGCs).^117^ RGC dendrites contain mitochondria closer to synapses than would be expected by chance.

As an essential factor for synaptic plasticity, mitochondrial architecture is modulated by synaptic activity.^90^ Long‐term potentiation (LTP) induction, a form of synaptic plasticity based on neuronal activity, stimulates Drp1, a major mitochondrial GTPase, and further mitochondrial fission in dendrites. Interestingly, it has been proven that inhibiting mitochondrial fission leads to an impaired rise in the calcium level of the mitochondrial matrix and, therefore, inhibits LTP in neurons.^125^ This LTP‐dependent mitochondrial fission also has intense behavioral manifestations like cocaine‐seeking behavior.^126^ In addition to bioenergetics mechanisms, mitochondria can modulate LTP in other ways.^90^ For example, calcium homeostasis in dendrites largely depends on ER‐mitochondria tethering. Without this successful tethering, calcium remains in the cytosol after neuronal activity and does not enter the mitochondrial matrix, leading to disrupted dendritic synaptic plasticity.^127^ Increased intracellular Ca^2+^ concentrations prevent mitochondrial mobility.^105^ Enhanced glutamate's activation of NMDA receptors at postsynaptic terminals results in increased Ca^2+^ influx via ionotropic glutamate receptors, which reduces mitochondrial mobility in cultured neurons.^128^, ^129^ It should be mentioned that other ATP‐gated receptor channels like P2X receptors are present in synapses^51^, ^130^ with calcium permeability greater than glutamate ionotropic receptors^50^ that control synaptotoxicity in a manner even more relevant than glutamate.^52^ These excitotoxic neuronal injury may also result in pathological changes in mitochondrial function.^104^

**FIGURE 2:**
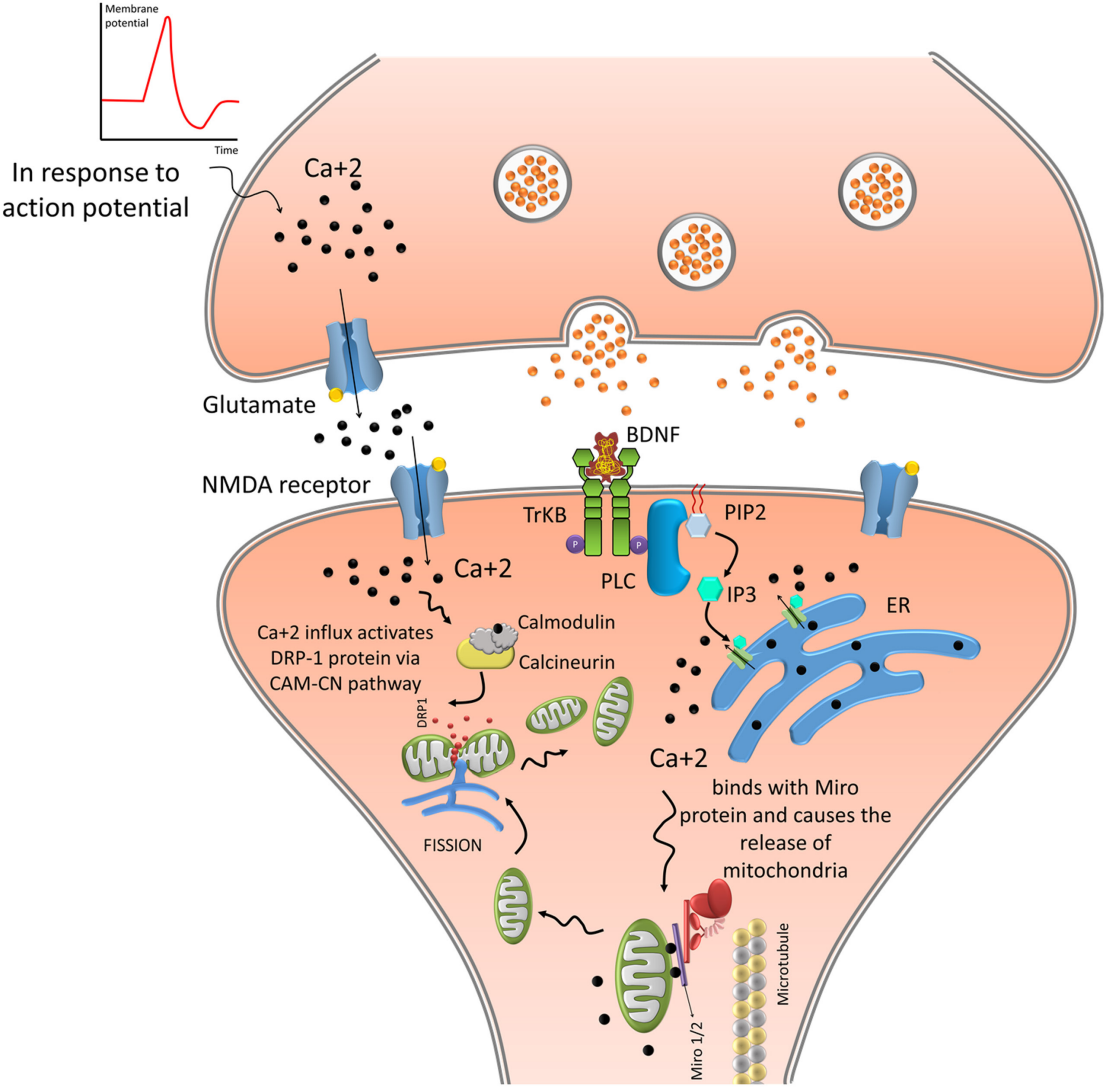
Mitochondrial localization and synaptic plasticity. At the presynaptic level, BDNF‐induced TrkB signaling stops the transport of mitochondria along the axons and promotes their docking at presynaptic sites by a Ca^2+^‐dependent mechanism involving the adaptor protein Miro1. The signaling mechanism operating downstream of TrkB receptors to regulate the transport of mitochondria is mediated by an increase in the [Ca^2+^] I through PI3K and PLC‐ɣ signaling pathways. Additionally, BDNF‐signaling controls mitochondrial transport and localization. More mitochondria build up at presynaptic sites due to the BDNF‐induced mitochondrial halting, followed by improved synaptic transmission. At the postsynaptic level, BDNF induces the delivery of AMPA and NMDA receptors to the synapse.

An adequate intracellular mitochondrial distribution is essential for optimum neuronal cell functioning. Loss of synapses and dendritic spines arises from molecular alterations in Drp1 (dynamin‐related protein‐1) and OPA1 (optic atrophy 1), leading to a decrease in the number of dendritic mitochondria. Conversely, an increase in the number or activity of dendritic mitochondria enhances the quantity and plasticity of synapses and spines. Therefore, the distribution of mitochondria in the dendrites is crucial and restrictive for maintaining synapses^122^ (Figure 2).

## 4 MITOCHONDRIAL TRANSPORT TO GUARANTEE NORMAL SYNAPTIC AND NEURONAL FUNCTION

Synaptic activity and mitochondrial transport and distribution in neurons are closely connected. The proper functioning of axons and synapses depends on the efficient transport of mitochondria and their settlement at the synapses. Like any other transportation system, mitochondrial transport requires a track (like microtubules and their associated proteins such as Tau), a motor (kinesin motors for anterograde directions and dynein motors for retrograde directions), and a cargo (which is the mitochondria itself).^131^ The microtubules act as highways through which microtubule‐associated proteins, such as Tau and motor proteins, can transport membrane‐bound organelles, such as mitochondria, into synapses.^132^, ^133^ Previous studies on both in vitro and in vivo models of AD revealed that the normal mitochondrial localization and distribution can be hindered by hyperphosphorylation of tau,^134^, ^135^, ^136^, ^137^ which can ultimately cause synaptic loss and axonal dysfunction.^133^, ^138^, ^139^ Hyperphosphorylated and truncated tau have been shown in these studies to increase the number of stationary mitochondria.^140^, ^141^ Furthermore, research utilizing various tau disease models demonstrates an increase in inter‐microtubular distance, which may account for reduced mitochondrial mobility.^136^ At the same time, neuronal models expressing pathogenic variants of tau exhibit a decrease in anterograde transport and an increase in retrograde transport that may lead to perinuclear accumulation of mitochondria.^135^, ^142^

Mitochondria are divided into two populations based on their localization: synaptic and non‐synaptic mitochondria.^91^ Determining precisely what signals draw mitochondria to particular neuron regions to localize them needs to be determined. Both synaptic and non‐synaptic mitochondria are synthesized in the soma, so mitochondrial transmission is required to reach their site of action. Non‐synaptic mitochondria are distributed throughout the cell body and in neuronal prolongations, whereas synaptic mitochondria are only found in synapses, both at the pre‐ and postsynaptic regions.^143^

Additionally, the findings of a seminal study conducted by Brown et al. indicate cortical synaptosomes mitochondria from rat had a higher tendency to undergo mitochondrial permeability transition (mPT) when exposed to additional Ca^2+^ in comparison with mitochondria from a non‐synaptosomal segment. The disparity observed is not attributable to mitochondrial energy or Ca^2+^ load variations. Instead, it may be attributed to the predominantly neuronal source of synaptic mitochondria compared to the mixed cellular origin of non‐synaptic mitochondria or the distinct roles played by synaptic and non‐synaptic mitochondria. The changes in Ca^2+^ buildup may be due to the isolated character of synaptic mitochondria, as opposed to the threads and clusters present in other cellular regions, alterations in cyclophilin D levels, or the increased age and cumulative oxidative damage to synaptic mitochondria.^85^ So, it is unsurprising that synaptic mitochondria exhibit different functional properties from non‐synaptic mitochondria because of further energy demand to maintain synaptic activity. For instance, synaptic mitochondria produce more peroxide than non‐synaptic mitochondria.^144^ These disparities between these two mitochondrial populations appear to be amplified as people age. Synaptic mitochondria exhibit lower oxidative capacity and increased vulnerability to calcium overload versus non‐synaptic mitochondria.^145^ Also, aging causes a premature mitochondrial enlargement in the hippocampal synaptic mitochondria, contributing to hippocampus‐dependent memory loss.^146^

The pattern of neuronal activity and external signals interacting with surface receptors control the highly controlled process of transporting mitochondria along neurites. These signals work by regulating both the activity and distribution of mitochondria. Clustered mitochondria at the synapses are distinct from their non‐synaptic counterparts, displaying unique morphological proteomic and Ca^2+^ handling and heightened susceptibility to oxidative damage.^62^

Increased biogenesis, a change in the ratio of docked to moving mitochondria, or a combination of the two may be responsible for the rise in the bi‐directional transport of mitochondria associated with synapse creation. Synapse development increases the generation of new mitochondria, improving mitochondrial transport over time but not changing stationary mitochondria density.^103^

Proper synthesis of mitochondria and their particular retrograde and anterograde transport along axons is essential for mitochondrial turnover.^132^ There are many static mitochondria at or near neuronal terminals to guarantee rapid neuronal firing. In the case of repetitive neuronal firing or prolonged energy demand, mitochondria can also be recruited in the sites.^122^, ^128^ In addition, old and dysfunctional mitochondria can over‐produce ROS, toxic agents for the cell. These dysfunctional mitochondria can have different destinies: (1) they may be repaired by fusing with new mitochondria that have just been transported from the soma, (2) they can be taken back to the soma for degradation and mitophagy, or (3) be degraded through in situ mitophagy in distal neuronal axons.^40^ Therefore, efficient transportation machinery is required to preserve cells from damage. To date, the most important mitochondrial transport system encompasses the kinesin heavy chain (KHC). Also, KIF5A and KIF5C are specific to neurons.^105^

When glutamate is applied acutely or when the calcium ionophore calcimycin is used to raise neuronal Ca^2+^ levels, mitochondrial transport is affected in neurons,^105^, ^129^ leading to recruited new mitochondria to synapses.^104^ Due to the dynamic nature of brain activity patterns, effectively managing mitochondrial mobility is vital for promptly redistributing mitochondria to different locations to meet the increased metabolic demands. On the other hand, a temporary halt in mitochondrial movement can be beneficial, as it allows them to be properly positioned, enhancing their ability to improve neuronal function.^147^ With increasing cytosolic Ca^2+^ concentration, the binding of Ca^2+^ to Miro leads to an immediate and transient structural alteration in the KHC/Milton/Miro complex. This structural change temporarily stops mitochondrial movement by separating the entire complex from microtubules^105^ or KHC from the mitochondria. On the other hand, as the Ca^2+^ concentration decreases, Ca^2+^ separates from Miro, and the complex re‐binds mitochondria to the microtubules and resumes their movement. This sensitivity of mitochondrial motility to Ca^2+^ concentration allows cells to locate mitochondria smartly in areas with high metabolic demand or low ATP concentrations (such as growth cones and postsynaptic specializations). For example, activated glutamate receptors recruit mitochondria in response to increased Ca^2+^ influx to uptake Ca^2+^ and prevent neurotoxicity.^104^, ^105^ In addition, studies have shown that BDNF can inhibit mitochondrial motility by stimulating Ca^2+^‐Miro binding in cultured hippocampal neurons, leading to mitochondria accumulation at presynaptic locations. Additionally, BDNF‐signaling controls mitochondrial transport and localization. More mitochondria build‐up at presynaptic sites due to the BDNF‐induced mitochondrial halting, followed by improved synaptic transmission in cultured hippocampus neurons.^148^ In areas that depend mainly on energy demand and Ca^2+^‐buffering ability, mitochondrial docking maintains the necessary number of stationary mitochondria.

As mentioned above, other factors, including mitochondrial fusion/fission machinery, affect mitochondrial transport. A physical interaction exists between mitochondrial fusion factors mitofusins (Mfn1 and Mfn2) that facilitates outer membrane fusion, Opa1 controls inner membrane fusion,^149^ and RhoT/Trak complex (RhoT is a membrane anchor protein, and Trak1 and Trak2 are its motor adaptors). It has been shown that in vitro and in vivo inhibition of mitofusins in neurons can significantly impair both the retrograde and anterograde transport.^150^ Drp1 is another fission protein that plays a vital role in mitochondrial transport. Drp1 interacts with the dynein–dynactin complex and modulates dynein‐based retrograde transport.^151^ MFF, FIS1, MiD49 (Scmr7), and MiD51 (Scmr7L) are the four currently recognized Drp1 receptors. Their relative role in Drp1‐dependent fission is still being discussed.^152^ According to Fukumitsu et al.^153^ study, inhibition of Drp1 impairs mitochondrial translocation toward dendrites in Purkinje cells. In addition, Berthet et al.^154^ reported that Drp1 plays a role in the mitochondrial distribution in the nerve terminals of dopaminergic neurons. Drp1 loss reduces axonal mitochondria in the midbrain and also mitochondrial division. Syntabulin is an adaptor protein that binds to mitochondria to help the anterograde movement along axons. Research revealed that siRNA suppression of syntabulin in the neurons decreased the distribution of mitochondria in dendrites and axons and led to synaptic dysfunction, including slower recovery, impaired transmission at a high rate, decreased basal activity, impaired short‐term plasticity, and SV depletion.^155^

Ohno et al.^156^ designed a time‐lapse imaging study to track mitochondrial mobility and distribution in myelinated CNS axons using cerebellar organotypic slice cultures. This study revealed decreased mitochondrial movement and nodal and paranodal axoplasm accumulation following repetitive axonal firing.

## 5 MITOCHONDRIAL DYSFUNCTION IN NEURODEGENERATIVE DISEASES

Some studies discuss that the Aβ deposition triggers mitochondrial dysfunction,^157^, ^158^ while others believe that mitochondrial dysfunction is upstream of the Aβ cascade.^159^ Several evidence shows that mitochondrial dysfunction happens earlier than the Aβ production in AD animal models.^160^, ^161^ Also, NFTs and Aβ accumulations impair the mitochondrial function and integrity in in vivo and in vitro models.^162^, ^163^ Additionally, studies on AD models have shown that Aβ‐mediated pathways can cause mitochondrial Ca^2+^ overload and further production of superoxide radicals and pro‐apoptotic mitochondrial proteins such as caspases and cytochrome C, all leading to cell death and neurodegeneration.^164^ Besides, soluble Aβ enhances mitochondrial Ca^2+^, which causes mPTP activation, ∆Ψm reduction, and caspase activation.^165^

The accumulation of mitochondria within dendritic spines, known as dendritic beading, is a phenomenon observed under conditions of extreme excitatory synaptic activity and glutamate toxicity as seen in pathological conditions like AD, in which accumulation of Aβ exacerbates glutamate excitotoxicity. During glutamate excitotoxicity, calcium influx induces mitochondrial depolarization, while sodium influx triggers an unsustainable rise in ATP demand via Na+, K + ‐ATPase activity. This results in decreased ATP levels, intracellular sodium accumulation, and subsequent water influx, leading to microtubule depolymerization, mitochondrial collapse, and dendritic beading.^166^ Dendritic beading suggests a concerted effort by neurons to cope with the increased metabolic demands and oxidative stress associated with excitotoxicity. However, this compensatory mechanism may ultimately contribute to mitochondrial‐dependent irreversible synaptic dysfunction and neurodegeneration, highlighting the intricate interplay between mitochondrial dynamics, dendritic structure, and glutamatergic neurotransmission in the pathogenesis of neurological disorders.

Previous studies have described a definite link between phosphorylated tau and mitochondrial dysfunction, although the causality remains unknown.^167^ Overexpression of mutant tau variants that promote phosphorylation is associated with aberrant distribution of mitochondria and their dysfunction in neurons from mice models of tauopathies and AD.^135^, ^168^ Tau hyperphosphorylation may impact mitochondrial function at three levels: transport, dynamics or morphology, and bioenergetics.^168^

ApoE‐ɛ4 variant is a prominent genetic factor associated with late‐onset AD and is believed to contribute to the decline in brain function associated with AD.^169^ The ApoE‐ɛ4 determines the acute neurological outcome of patients by interacting with the mitochondrial genome.^170^ A study conducted by Yin et al.^171^ reported that ApoE‐ɛ4 reduced the dynamics and biogenesis of mitochondria. Their finding indicated that ApoE‐ɛ4 hindered the process of biogenesis of mitochondria by decreasing the levels of two key factors: sirtuin 3 (SIRT3), a protein found in mitochondria that helps preserving the metabolic homeostasis, and PGC‐1α, a member of the PGC family that acts as a transcriptional activator and controls the expression of SIRT3. Additionally, their research indicated that ApoE‐ɛ4 may impact synaptic plasticity by regulating the activity of mitochondrial proteins.

Mitochondrial biogenesis in neurons can take several hours to a few days, depending on factors such as the type of neurons, the state of cellular health, and external stimuli. The transportation speed of mitochondria in neurons is about 0.5 μm/s,^172^ which means that in big neurons, it would take a few days for a newly generated mitochondrion to get from the cell body to the axon tip. Synaptic plasticity occurs from several seconds to several days after the induction stimulus. The early synaptic plasticity phase does not seem dependent on mitochondrial biogenesis. However, recently, it has been shown that mitochondria are also produced locally at synapses in response to neuronal activity. Indeed, critical RNAs necessary for synthesizing mitochondrial proteins are present in the presynaptic terminal. Increased neuronal activity prompts their translation multiple times, facilitating in situ mitochondrial synthesis.^173^ As reported in previous studies, synaptic plasticity can be divided into short‐term synaptic plasticity (STSP),^174^ which lasts for milliseconds to minutes, and long‐term synaptic plasticity (LTSP),^175^, ^176^ which lasts for at least tens of minutes to hours or longer. Eventually, the time required for long‐term synaptic plasticity (LTSP) would encompass the time needed for mitochondrial biogenesis. This suggests that local mitochondrial biogenesis supports the initial phase of synaptic plasticity, while mitochondrial biogenesis in the soma supports the later stages of synaptic plasticity.

As stated earlier, basic and clinical research studies have established a significant association between mitochondrial impairment, further synaptic dysfunction, and the extent of memory loss in AD. It is worth mentioning that noticeable alterations in synapses have been observed in the neocortex of patients, even in the initial stages of AD or with mild cognitive impairment (MCI), despite the presence of modest pathological changes.^177^ These data confirm the hypothesis that AD is a condition characterized by impaired synaptic function.^17^

Recent reports mainly focus on the impact of mitochondrial dysfunction and immunological responses in the development of AD, suggesting that dysfunctional mitochondria release mitochondrial components, such as mtDNA, through several pathways that trigger inflammatory reactions via pattern recognition receptors (PRRs) that lead to the initiation of a cascade of intracellular signaling pathways leading to neuronal death.^178^ The significance of mitochondrial dynamics in AD has also been noticeable in recent years.^179^ Altered mitochondrial dynamics have been seen in AD, characterized by increased mitochondrial fission and decreased fusion. This imbalance in mitochondrial dynamics can result in the accumulation of defective mitochondria, thereby exacerbating both mitochondrial and synaptic dysfunction.^166^, ^180^, ^181^

In the pathogenesis of AD, there appears to be a complex interplay between the accumulation of Aβ and phosphorylated tau proteins and mitochondrial dysfunction. Aβ and phosphorylated tau have disrupted mitochondrial function, leading to mitochondrial disorder. Conversely, impaired mitochondrial function exacerbates the accumulation of Aβ and phosphorylated tau, creating a vicious cycle. This dysregulated cycle ultimately contributes to neuronal damage and cell death, hallmark features of AD. However, the precise sequence of events in this pathological cascade remains elusive, presenting a conundrum reminiscent of the age‐old question of which came first, the chicken or the egg! Understanding the temporal relationship between these events is crucial for unraveling the underlying mechanisms of AD and developing effective therapeutic interventions.

## 6 CONCLUSION

In summary, mitochondria serve as fundamental components in synaptic activity. Their role in supplying energy demand, buffering calcium and vesicle recycling contributes significantly to neurotransmission. Notably, mitochondria in the presynaptic terminal exhibit distinct morphological features compared to those in the postsynaptic terminal. The mechanism underlying this morphological adaptation across different neuronal compartments remains unclear. Mitochondrial plasticity, the process by which mitochondria adapt to increased network activity, raises compelling questions about its connection to synaptic plasticity. Untangling the complex interplay between mitochondrial and synaptic plasticity is pivotal for comprehending normal cellular processes and the pathophysiology of neurodegenerative diseases. Further research in this area holds promise for developing targeted interventions to preserve synaptic function and mitigate the impact of mitochondrial dysfunction in AD and other related conditions.

## 7 FUTURE PERSPECTIVES

This review underscores mitochondria's significant role in basic synaptic activity and plasticity. However, the sequence of changes—whether synaptic alterations lead to mitochondrial changes or vice versa—remains unclear. Future research should aim to elucidate this relationship. Advanced imaging techniques should be employed to investigate mitochondrial changes before and after synaptic plasticity events. This approach can help determine whether synaptic activity induces mitochondrial adaptations or if mitochondrial dynamics drive synaptic modifications. Additionally, it is crucial to identify which regions of the neuron (soma, presynaptic, or postsynaptic compartments) are most affected in pathological conditions such as Alzheimer's disease and Parkinson's disease. Another promising avenue is exploring gene therapy's potential to correct mitochondrial defects. Understanding whether restoring mitochondrial function can mitigate synaptic dysfunction in neurodegenerative diseases could open new therapeutic strategies. Evaluating basic mitochondrial activities, including bioenergetics, biogenesis, and quality control in healthy and diseased states, can help us better understand the role of mitochondria in physiological and pathological conditions. This knowledge can generate new therapeutic methods, such as medications that modify mitochondrial fission and fusion to restore balance in damaged neurons and therapies that improve mitochondrial transport to minimize degeneration and increase regeneration. Identifying early signs of mitochondrial imbalance in neurodegenerative disorders may also open up new paths for intervention, perhaps protecting against mitochondrial dysfunction and preventing the onset or progression of these ailments. By focusing on these study directions, we can better understand mitochondrial dynamics and their impact on neuronal function, potentially leading to more effective treatments for neurodegenerative illnesses and age‐related neuronal decline. Overall, clarifying the interplay between mitochondrial and synaptic plasticity will enhance our understanding of neurodegenerative disease mechanisms and guide the development of innovative treatments.

## CONFLICT OF INTEREST STATEMENT

None.

## Data Availability

Data sharing is not applicable to this article as no new data were created or analyzed in this study.
